# Correction of Supernumerary Tooth‐Associated Anterior Crossbite in Mixed Dentition Using a 2 × 4 Appliance

**DOI:** 10.1155/crid/1417850

**Published:** 2026-08-08

**Authors:** Prasanna Chandraseharan, Bavani Sinnumu Naidu, Thaarani Vijayakumar, S. Nagarajan M. P. Sockalingam

**Affiliations:** ^1^ Department of Paediatric Dentistry, Hospital Teluk Intan, Teluk Intan, Perak, Malaysia; ^2^ Department of Paediatric Dentistry, Hospital Tengku Ampuan Rahimah, Klang, Selangor, Malaysia, htar.moh.gov.my; ^3^ Department of Paediatric Dentistry, Women and Children Complex, Tengku Permaisuri Norashikin Hospital, Kajang, Selangor, Malaysia; ^4^ Department of Family Oral Health, Faculty of Dentistry, The National University of Malaysia (UKM), Kuala Lumpur, Malaysia, ukm.my

**Keywords:** 2 × 4 appliance, anterior crossbite, interceptive orthodontics, mixed dentition stage

## Abstract

Anterior crossbite is a common tooth alignment discrepancy seen among the paediatric population especially during the mixed dentition stage. There are many causes of anterior crossbite, and the presence of supernumerary tooth/teeth is one of them. Early interceptive intervention is essential to avoid the need for more complex orthodontic treatment later. This case report describes the management of a 10‐year‐old child who presented with anterior crossbite involving Teeth 21 and 31, leading to gingival recession of Tooth 31. The crossbite malocclusion was associated with a supernumerary tooth in the upper incisor region. Extraction of the supernumerary tooth was performed, and correction of the anterior crossbite was commenced with a 2 × 4 appliance. Adequate stability with a positive overjet was observed at the 6‐month review. The 2 × 4 orthodontic appliance may be considered a treatment option for correcting anterior crossbite in patients with poor compliance with removable appliance therapy.

## 1. Introduction

Anterior crossbite is defined as an abnormal incisor relationship of a tooth or teeth in which the maxillary incisor or incisors occlude lingually to the mandibular incisor tooth or teeth [1]. The prevalence of anterior crossbite ranges from 2.2% to 11.9%, which varies according to the study population, age and the criteria used to define anterior crossbite [2–4].

Factors that cause anterior crossbite can be categorised into skeletal, dental or functional. Skeletal causes can be attributed to hereditary influences or genetic factors that contribute to the discrepancy in the size of the maxilla and mandible. Skeletal discrepancy usually involves a segment of the maxillary and mandibular teeth that is in an abnormal alignment, where a portion of the maxillary teeth is at normal angulation but positioned posterior to the mandibular incisors. Dental crossbites often involve one or two teeth, and the affected teeth are either upright or reclined. The dental causes usually do not involve a significant discrepancy between the maxilla and mandible. Meanwhile, a functional crossbite involves premature contact between an opposing tooth or teeth that can cause abnormal deflection of the mandible upon closure to achieve a comfortable occlusion [5].

Dental anterior crossbites often need prompt intervention in the early stages of the mixed dentition. Failure to identify and treat these crossbites can lead to adverse consequences such as premature contact, enamel wear, gingival recession, loss of periodontal stability, loss of alveolar bone support and tooth mobility [5–7].

Numerous treatment modalities have been advocated for correcting anterior crossbites. However, selecting an appropriate treatment option depends on the aetiology of the crossbite, the patient′s age, patient compliance, parental motivation, eruption stage of the teeth, availability of space and cost of the treatment [5]. In skeletal crossbite cases, a combination of treatment with rapid maxillary expansion, reverse‐pull facemasks or Frankel III appliance may correct the discrepancies [5, 6]. Fixed, removable appliances, clear aligners, tongue blades and inclined planes can assist in correcting nonskeletal origin crossbites [8–13]. The chosen treatment modality should not only aim to correct the crossbite but also be cost‐effective, easily tolerated by patients and cause no harm to the surrounding soft tissue [11].

The 2 × 4 appliance is fixed, simple, easy to apply and well‐tolerated by patients to correct anterior crossbites in the mixed dentition stage [14]. The current case report highlights a case of anterior crossbite that was successfully corrected using the 2 × 4 fixed appliance. This technique provided fast and good long‐term stability with minimal patient compliance during clinical management.

## 2. Case Presentation

A 10‐year‐old boy was referred to the department to manage an extra tooth in the area of his upper front teeth. His parents were concerned about the crooked appearance of his teeth, which was getting worse over time. The boy was an irregular dental attendee with an unremarkable medical history.

The extraoral face assessment showed a symmetrical frontal appearance with a straight facial profile. Intraoral examination revealed that the boy was in his late mixed dentition stage with bilateral Angle′s Class I molar relationships. The boy′s oral hygiene was good, with a plaque score of 0.83 using the Simplified Oral Hygiene Index (OHI‐S) [15]. There was an erupted tuberculate‐shaped supernumerary tooth palatal to Tooth 11. There was crossbite with relation to Teeth 21 and 31, and median diastema of 2 mm. There was also a 2‐mm discrepancy of the lower midline to the left side. Tooth 31 was displaced labially with gingival recession at its labial aspect because of traumatic occlusion (Figure 1A,C). Besides that, occlusal interference was noted at the affected teeth region with premature contact upon closure, with deviation of the centric occlusion to the left resulting in crossbite occlusion. The cone beam computed tomography (CBCT) showed no other supernumerary tooth/teeth or abnormalities at the maxillary anterior region (Figure 2A–C). The treatment plan for extraction of the supernumerary tooth, followed by a 2 × 4 appliance for the correction of anterior crossbite was planned after discussing with the parents.

**Figure 1:**
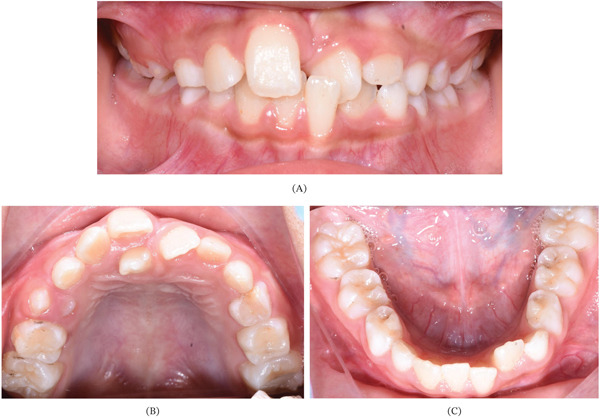
Pretreatment clinical photographs. (A) Anterior view: mesiopalatal rotation and crossbite of the maxillary left central incisor with mandibular left central incisor. (B) Maxillary occlusal view: erupted supernumerary at palatal of the maxillary right central incisor and mesiopalatal rotation of the maxillary left central incisor. (C) Mandibular occlusal view: mandibular left central incisor displaced labially and mandibular left lateral incisor displaced lingually from the mandibular arch.

**Figure 2:**
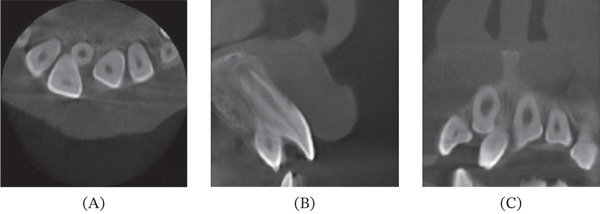
Cone beam computed tomography (CBCT) of (A) axial view, (B) sagittal view and (C) coronal view revealed a tuberculate‐shaped supernumerary at the palatal of the maxillary central incisor with a straight root. No other supernumeraries noted.

The patient showed great anxiety when local anaesthesia was to be administered; thus, extraction of the supernumerary tooth was performed under nitrous oxide oxygen inhalation sedation after taking informed written consent from the patient′s mother. After 2 weeks, with satisfactory healing of the extraction site, the patient was set up with a 2 × 4 orthodontic appliance to correct his anterior crossbite. Bands were placed and cemented on the maxillary right and left first permanent molars (16 and 26). Four preadjusted edgewise brackets with 0.022 ^″^ slots were bonded to the maxillary permanent incisors (Teeth 12, 11, 21 and 22). At the first visit, 0.012 ^″^ round nickel‐titanium (NiTi) archwire was placed into the bracket and band slots and secured with elastic ties. The patient′s occlusion was raised by 2 mm using Type II glass ionomer cement on the mandibular primary second molars and permanent first molars (Figure 3). The NiTi archwire was changed monthly at an increasing thickness to 0.014 ^″^, followed by 0.016 ^″^ on the subsequent visit. After 3 months of treatment, there was positive overjet and derotation of Tooth 21, suggestive of successful anterior crossbite correction. Tooth 31 moved inwards and was noted to be well aligned in the arch. Besides that, the gingival health of Tooth 31 also had improved due to the absence of traumatic interference (Figure 4). The 0.016 ^″^ round NiTi archwire was changed to the 0.018 ^″^ round NiTi archwire and retained for another 4 weeks to increase stability before brackets and bands debonding. Further torquing of the roots was not performed because the roots were still immature, and excessive force may result in undesired root resorption. Furthermore, the primary aim of the treatment was to correct the crossbite discrepancy. At the 6‐month follow‐up visit, the incisors were stable, corrected with positive overjet and had improved gingival health (Figure 5A–C). Spontaneous closure of the remaining 2 mm of median diastema in the maxillary arch was expected to occur with the eruption of the permanent canines or will be managed at a later stage if spontaneous closure fails to take place.

**Figure 3:**
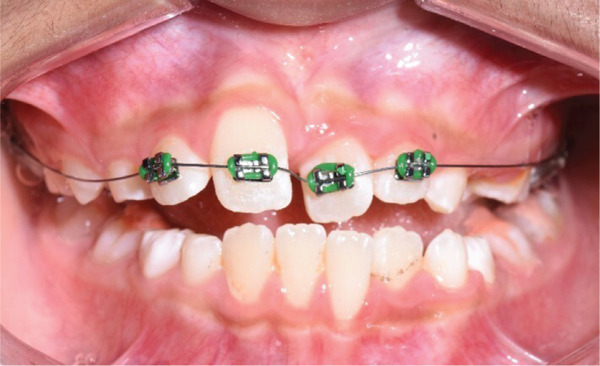
Clinical photograph during initial treatment: Bonding of four preadjusted edgewise brackets with 0.022 ^″^ slots to the maxillary permanent incisors with cementation of molar bands and attachment with 0.012 ^″^ round NiTi arch wire.

**Figure 4:**
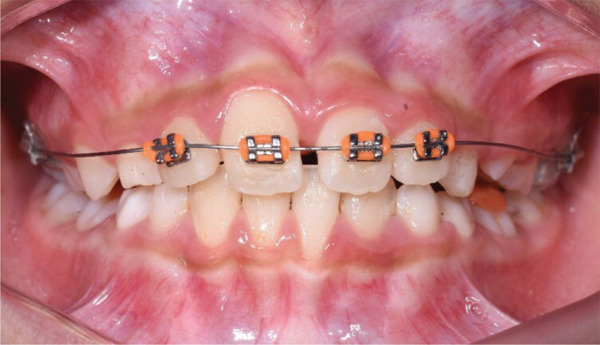
Clinical photograph during ongoing treatment. Post 3 months of initialising 2 × 4 appliance, noted positive overjet and correction of rotation and anterior crossbite.

**Figure 5:**
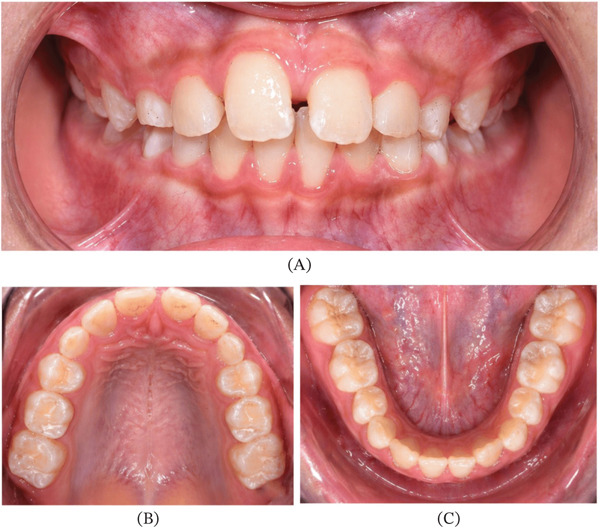
Six‐month follow‐up photographs. (A) Anterior view: correction of mesiopalatal rotation and anterior crossbite of the maxillary left central incisor with mandibular left central incisor. (B) Maxillary occlusal view: correction of the mesiopalatal rotation of the maxillary left central incisor. (C) Mandibular occlusal view: mandibular left central incisor moved inwards and appears well aligned in the arch.

## 3. Discussion

Crossbite correction is successful when a positive overjet is achieved with adequate overbite while holding the affected tooth in position. Dental‐related causes are the main contributors to anterior crossbite as compared with skeletal and functional aetiologies. Primary contributors of anterior crossbite include supernumerary tooth/teeth, odontomas, ectopic position of the permanent tooth germ, retained primary tooth, trauma to the primary predecessor, anomalies in tooth shape and size and arch length discrepancy. These pathologies and developmental anomalies often impede the eruption of the permanent tooth/teeth and, in some instances, may deflect from the normal tooth/teeth eruption path [5, 16].

Supernumerary teeth are a developmental anomaly characterised by increased tooth buds in primary or permanent dentition. Genetic factors, hyperactivity of the dental lamina or the tooth bud dichotomy can cause the development of supernumerary teeth [17]. Supernumerary teeth occur frequently in the anterior region, followed by the premolar region. The incidence is reported to be between 1.5 and 3.9% in permanent dentition with a male predilection [18]. Supernumerary teeth can be classified by their shape, number, location and morphology. The morphological types can be classified into conical, tuberculate, supplemental or odontoma. The conical variation is the most reported type, with 31%–75% incidence. The presence of supernumerary teeth often causes clinical consequences such as the development of caries, cysts, resorption of adjacent roots, retention of deciduous teeth, delayed eruption of permanent teeth and occlusal disturbances of developing dentition [17]. The presence of supernumeraries also causes displacement of the crown of the permanent incisors. This deflection of tooth/teeth may cause crowding of the anterior dentition, resulting in anterior crossbite and esthetic problems for the child [19]. As highlighted in this case report, an erupted tuberculate‐shaped supernumerary caused deflection in the eruption of the permanent incisor, resulting in an anterior crossbite. Removal of the obstacles is advocated before the correction of the crossbite.

CBCT is used in the current case because it is considered a better option than analogue two‐dimensional (2D) x‐rays for detecting supernumerary teeth in the midline/premaxilla. CBCT allows accurate localisation and assessment of the position and orientation of a supernumerary tooth in the buccolingual, mesiodistal and vertical planes, which is not possible with 2D radiographs. Furthermore, it eliminates superimposition of bony structures, such as the nasal floor, incisive canal and cortical plates, which may obscure detection of microdontic teeth, such as the mesiodens. CBCT also has greater sensitivity and specificity to detect minute changes, such as root resorption of the permanent incisors adjacent to the unerupted supernumerary, especially on the palatal root surfaces. CBCT also helps surgeons plan their surgery to minimise surgical trauma and avoid iatrogenic injuries to the vital permanent teeth [20].

Interceptive orthodontic treatment is carried out at the mixed dentition stage to resolve or prevent adverse occlusal abnormalities and to allow normal skeletal pattern development in growing children. The age of 8–11 years is an ideal period for interceptive orthodontic treatment because the roots are immature and have eruption potential during this period [11]. Treatment at an early stage is deemed necessary to prevent further detrimental consequences such as attrition of dentition, gingival recession, mobility, deviation of jaws and an increase in severity of malocclusion, which may necessitate more complex orthodontic procedures [5–7].

Treatment modalities offered to patients for anterior crossbite correction should be simple and noninvasive, involve less chairside time and require minimal patient compliance for quick crossbite correction and long‐term stability after correction [11]. Several treatment modalities address anterior crossbite cases, such as removable appliances, tongue blades, inclined planes, clear aligners and 2 × 4 appliances [8–13].

Removable orthodontic appliances are the primary treatment choice to correct anterior crossbites as they require less chairside time and are effective [11]. However, a higher compliance rate is needed to provide efficacious treatment. Furthermore, patients may experience speech difficulty due to bulky palatal coverage. Besides that, appliance loss, poor oral hygiene maintenance and appliance breakage can occur [21].

Tongue blade therapy is a viable option during the early stage of tooth development. The tongue blade must be placed at an angle between the affected teeth, and the patient is instructed to apply force in a controlled manner. However, this technique requires the patient′s cooperation and compliance to perform the exercise for at least 1–2 h per day for about 2–3 weeks continuously [8].

An acrylic inclined plane (Catlan′s appliance) can treat multiple teeth in crossbite without requiring the patient′s compliance as the plane caps will be cemented on the lower incisors at a 45° angle to the occlusal plane. However, speech and mastication of the child will be affected during the treatment period [22]. The removable option of an inclined plane would be the Bruckl appliance, a simple, functional appliance used when an anterior crossbite involves several teeth and with vertical overbite approximately 1/2 or more [23].

Clear aligners have also been reported in the literature to correct anterior crossbites. The aligners are less bulky, well‐tolerated, easy to care for and aesthetically pleasing. However, this technique requires multiple aligners because the activation forces decrease and plateau after 5 days of use for each aligner, which increases treatment costs [10].

Fixed orthodontic treatment with 2 × 4 appliances is an excellent alternative for cases requiring complex tooth movement beyond simple tipping. Besides, 2 × 4 appliances also overcome patients′ compliance issues that are commonly seen with removable appliances, especially among paediatric patients and further result in failure of achieving crossbite correction, requiring an alternative treatment approach [24]. The appliance involves the placement of stainless‐steel bands with attached tubes on the first permanent molars, bonded brackets on the maxillary permanent incisors and continuous light archwire. The archwire will be inserted into the attached tubes on the stainless‐steel bands and the bonded brackets. This appliance corrects malaligned teeth in a controlled manner in all three‐dimensional directions, including tipping, rotation, torque and vertical movements, ensuring fast and predictable outcomes. Furthermore, the 2 × 4 appliances do not restrict mandibular growth or normal jaw function, allow normal speech, mastication and oral hygiene care, and are more psychologically and socially acceptable to children than bulkier removable appliances. Therefore, good patient selection, with motivated oral hygiene care, especially during the mixed dentition stage, can yield a quick and desirable outcome with the 2 × 4 appliance compared with other alternatives in growing patients [14].

Systematic reviews comparing fixed and removable appliances have shown that both have high success rates [25, 26]. However, anterior crossbite correction with fixed appliances has a significantly shorter treatment time at an average of 5.5 months and is more cost‐effective [27]. The increased treatment time for removable appliances could be due to patient noncompliance with appliance wear. At the same time, the higher total cost of removable appliance therapy could be attributed to increased treatment time, laboratory charges and increased expenses on repairing fractured clasps or acrylic plates [26]. No significant differences were reported in quality of life, pain intensity or long‐term stability between the two appliances [25, 26].

## 4. Conclusions

The current case demonstrated the usage of the 2 × 4 appliance to correct the anterior crossbite caused by the supernumerary tooth. With the 2 × 4 appliance, patient‐dependent compliance is not required, yet a rapid correction is achieved. With the correction of malocclusion, the patient′s esthetic concerns are also addressed promptly. The alignment of teeth is well established, with harmonious stability between the dental and soft tissue components and optimal intercuspation. This technique does not necessarily prevent future orthodontic treatment, but may reduce the complexity and duration of any subsequent treatment required.

## Funding

No funding was received for this manuscript.

## Ethics Statement

Ethical approval was obtained from the National Medical Research Register (NMRR) of the Ministry of Health Malaysia (NMRR ID‐24‐03580‐H50).

## Consent

Written informed consent was obtained from the patient′s parents for documentation and publication.

## Conflicts of Interest

The authors declare no conflicts of interest.

## Data Availability

The data that support the findings of this study are available on request from the corresponding author. The data are not publicly available due to privacy or ethical restrictions.
